# Metagenome-Assembled Genomes of 12 Bacterial Species from Biofouled Plastic Fabrics Harbor Multiple Genes for Degradation of Hydrocarbons

**DOI:** 10.1128/MRA.01458-20

**Published:** 2021-05-06

**Authors:** Osman Radwan, Oscar N. Ruiz

**Affiliations:** a Environmental Microbiology Group, University of Dayton Research Institute, Dayton, Ohio, USA; b Fuels and Energy Branch, Aerospace Systems Directorate, Air Force Research Laboratory, Wright-Patterson AFB, Ohio, USA; Indiana University, Bloomington

## Abstract

We report the metagenome-assembled genomes (MAGs) of 12 different bacterial species recovered from environmental microbiomes associated with biofouled plastic fabrics. The MAGs have estimated sizes of 2.53 to 7.66 Mb with 3,229 to 9,289 proteins, 26.20% to 99.1% genome completeness, 48.9% to 72.6% G+C content, and multiple genes for hydrocarbon degradation.

## ANNOUNCEMENT

Several bacterial species have been shown to degrade polymers and hydrocarbon fuel (1–7). The fuel and plastic biodegradation activity of these species is attributed to multiple hydrocarbon-degrading enzymes (1–5) and hydrolytic enzymes (6, 7), respectively. In this study, bioinformatic tools were used for genome assembly and annotation of 12 bacterial species recovered from a shotgun metagenomic library of environmental microbiomes associated with biofouled plastic fabrics (8).

As explained by Radwan et al., tent shelter plastic fabric samples exposed for 14 months to the Panama jungle were retrieved from the location site and stored refrigerated at 4°C (8). Samples were cut into 0.5-cm^2^ pieces for DNA extraction using the Qiagen DNeasy UltraClean kit (catalog number 12224-250) (8). A PrepX DNA library kit and an Apollo 324 next-generation sequencing (NGS) automatic library prep system were used to construct the DNA libraries (WaferGen, Fremont, CA). An Illumina HiSeq 2000 instrument was used to sequence the DNA libraries, generating 161,537,275,209 raw reads with an average length of 100 bp (8). For quality control, Trimmomatic 0.36 (9) was used to remove raw reads with average quality below 15 and those with a length less than 50 bp. Sequence assembly and binning of the different population-level genomes were conducted with an in-house bioinformatic pipeline comprising multiple bioinformatic programs (10) with default parameters unless otherwise noted. The bioinformatic pipeline sequentially applied, as described next, the programs BBtools (https://jgi.doe.gov/data-and-tools/bbtools/), MEGAHIT (11), Bowtie 2 (12), SAMtools (13), Pileup (13), AWK (14), MaxBin (15), SSPACE (16), GapFiller (17), RepeatMasker (18), Prodigal (19), ABySS (20), and HMMER (21). BBtools was used for sorting paired-end reads and normalization to ensure compatibility before sequence assembly using MEGAHIT with the options minimum contig length of 200 bp and meta-sensitive. The produced contigs from MEGAHIT were subjected to Bowtie 2 for mapping raw reads to contigs and to create BAM files that were converted to SAM files using SAMtools to generate the coverage matrix and abundance files in Pileup and AWK, which were finally used in MaxBin, with the options minimum contig length of 2,000 and depth of 2, for binning of individual genomes.

An assembly improvement process and a sequence gap-filling process for the 12 bacterial genomes (Table 1) were performed using SSPACE and GapFiller, respectively. The masked genome sequences from RepeatMasker were used in Prodigal to annotate the function of genes. The completeness of the different genomes was extracted from the MaxBin output and ranged from 26.20% for Actinomycetospora chiangmaiensis to 99.1% for Mucilaginibacter polytrichastri. ABySS was used to calculate the genome sizes, *L*_50_ values, and G+C contents, which ranged from 2.53 to 7.66 Mb, 3 to 510 contigs, and 48.9% to 72.6%, respectively (Table 1). The proteins potentially involved in hydrocarbon degradation and polymer hydrolysis (Table 1) were identified with HMMER against the Pfam database with an E value of 0.001. The number of identified proteins for each pathway varied among species, with the Gordonia polyisoprenivorans and Williamsia herbipolensis genomes containing the highest numbers of protein-coding genes for both alkane and aromatic degradation (Table 1). Similarly, these two genomes contained a large number of hydrolase and efflux pump genes, which have been associated with resistance to toxic compounds (22). In total, 11 of the 12 bacterial genomes, all except Actinomycetospora chiangmaiensis, contained genes encoding hydrolases. The information derived from the assembly and annotation of the 12 metagenome-assembled genomes (MAGs) supports the ability of these bacterial species to degrade hydrocarbons (1–5) and, potentially, plastics (6, 7).

**TABLE 1:** Accession number, general statistics of metagenomic-assembled bacterial genomes, and number of genes involved in hydrocarbon degradation, polymer hydrolysis, and efflux pumps from each bacterial genome^*a*^

Code	Genome	Phylum	GenBank accession no.	Size (bp), coverage (×)	*L*_50_	*N*_50_ (bp)	No. of proteins	Completeness (%)	No. of degradation genes	No. of hydrolase genes	No. of MFS, no. of ABC^*b*^
E08	Methylobacterium mesophilicum	*Proteobacteria*	JADCRT000000000	3,992,127, 200	3	510,122	3,873	98.10	8	2	35, 141
E01	*Williamsia* sp.	*Terrabacteria*	JADCRZ000000000	7,661,838, 105	19	127,300	5,811	95.30	1	30	50, 76
F07	Mucilaginibacter polytrichastri	*Bacteroidetes*	JADCRU000000000	5,102,915, 157	39	36,208	4,843	99.10	0	10	39, 62
F05	Williamsia herbipolensis	*Terrabacteria*	JADCRY000000000	4,041,607, 279	136	7,953	4,076	54.20	33	12	65, 74
C12	Jatrophihabitans endophyticus	*Actinobacteria*	JADCRS000000000	3,929,516, 256	156	7,123	5,770	88.80	7	2	100, 75
F04	Gordonia polyisoprenivorans	*Terrabacteria*	JADCRR000000000	5,331,221, 198	153	10,270	5,333	86.00	53	26	104, 103
C15	*Caulobacter sp.*	*Proteobacteria*	JADCRP000000000	3,614,289, 278	203	4,973	4,184	70.10	4	3	66, 50
C10	Gluconacetobacter diazotrophicus	*Proteobacteria*	JADCRQ000000000	3,858,963, 260	238	5,084	4,157	50.50	2	1	89, 27
F33	*Acetobacter sp.*	*Proteobacteria*	JADCRW000000000	2,529,106, 446	245	2,672	2,780	32.70	0	1	35, 23
D02	Terriglobus roseus	*Acidobacteria*	JADCRX000000000	3,673,883, 304	483	2,489	3,179	33.60	6	5	22, 16
E11	Parafilimonas terrae	*Bacteroidetes*	JADCRV000000000	7,593,549, 105	499	4,441	8,697	55.10	18	1	145, 165
E07	Actinomycetospora chiangmaiensis	*Terrabacteria*	JADCRO000000000	5,278,631, 152	512	3,036	6,417	26.20	9	0	118, 111

a The Pfam database with an E value of 0.001 was used for functional annotation.

b MFS, major facilitator superfamily efflux pumps; ABC, ABC transporters.

### Data availability.

The raw metagenomic sequence reads and MAGs were deposited at DDBJ/ENA/GenBank under the BioProject accession number PRJNA656514 with BioSample accession numbers SAMN15786575 to SAMN15786586 and SRA accession numbers SRX9364069 to SRX9364074. The individual accession numbers of the MAGs are provided in Table 1.
